# WHO cardiovascular disease risk prediction model performance in 10 regions, China

**DOI:** 10.2471/BLT.22.288645

**Published:** 2023-02-01

**Authors:** Songchun Yang, Yinqi Ding, Canqing Yu, Yu Guo, Yuanjie Pang, Dianjianyi Sun, Pei Pei, Ling Yang, Yiping Chen, Huaidong Du, Dan Schmidt, Rebecca Stevens, Derrick Bennett, Robert Clarke, Junshi Chen, Zhengming Chen, Liming Li, Jun Lv, on behalf of the China Kadoorie Biobank Collaborative Group

**Affiliations:** a Department of Epidemiology and Biostatistics, Peking University Health Science Center, 38 Xueyuan Road, Beijing 100191, China.; b Fuwai Hospital, Chinese Academy of Medical Sciences, Beijing, China.; c Peking University Center for Public Health and Epidemic Preparedness & Response, Beijing, China.; d Medical Research Council Population Health Research Unit, University of Oxford, Oxford, England.; e Nuffield Department of Population Health, University of Oxford, Oxford, England.; f China National Center for Food Safety Risk Assessment, Beijing, China.

## Abstract

**Objective:**

To validate the World Health Organization (WHO) non-laboratory-based cardiovascular disease risk prediction model in regions of China.

**Methods:**

We performed an external validation of the WHO model for East Asia using the data set of China Kadoorie Biobank, an ongoing cohort study with 512 725 participants recruited from 10 regions of China from 2004–2008. We also recalculated the recalibration parameters for the WHO model in each region and evaluated the predictive performance of the model before and after recalibration. We assessed discrimination performance by Harrell’s C index.

**Findings:**

We included 412 225 participants aged 40–79 years. During a median follow-up of 11 years, 58 035 and 41 262 incident cardiovascular disease cases were recorded in women and men, respectively. Harrell's C of the WHO model was 0.682 in women and 0.700 in men but varied among regions. The WHO model underestimated the 10-year cardiovascular disease risk in most regions. After recalibration in each region, discrimination and calibration were both improved in the overall population. Harrell’s C increased from 0.674 to 0.749 in women and from 0.698 to 0.753 in men. The ratios of predicted to observed cases before and after recalibration were 0.189 and 1.027 in women and 0.543 and 1.089 in men.

**Conclusion:**

The WHO model for East Asia yielded moderate discrimination for cardiovascular disease in the Chinese population and had limited prediction for cardiovascular disease risk in different regions in China. Recalibration for diverse regions greatly improved discrimination and calibration in the overall population.

## Résumé

**Objectif:**

Valider le modèle de prévision hors laboratoire des risques de maladies cardiovasculaires de l'Organisation mondiale de la Santé (OMS) dans plusieurs régions de Chine.

**Méthodes:**

Nous avons effectué une validation externe du modèle de l'OMS pour l'Asie orientale en nous fondant sur l'ensemble de données de la China Kadoorie Biobank, une étude de cohorte en cours qui compte 512 725 participants recrutés dans dix régions de Chine entre 2004 et 2008. Nous avons également recalculé les paramètres de recalibrage du modèle de l'OMS dans chaque région, et mesuré les performances prévisionnelles de ce dernier avant et après recalibrage. Enfin, nous avons évalué ses capacités de discrimination selon l'index C de Harrell.

**Résultats:**

Nous avons inclus 412 225 participants âgés de 40 à 79 ans. Sur un suivi médian de 11 ans, 58 035 cas de maladies cardiovasculaires ont été enregistrés chez les femmes et 41 262 chez les hommes. Le modèle de l'OMS affichait un index C de Harrell de 0,682 pour les femmes et de 0,700 pour les hommes, mais variait d'une région à l'autre. En outre, il sous-estimait les risques de maladies cardiovasculaires sur dix ans dans la plupart des régions. Un recalibrage pour chacune d'elles a permis d'optimiser la discrimination et le calibrage pour la population dans son ensemble. L'index C de Harrell est passé de 0,674 à 0,749 pour les femmes et de 0,698 à 0,753 pour les hommes. Le ratio entre cas prévus et cas observés avant et après recalibrage s'élevait à 0,189 et 1,027 chez les femmes, contre 0,543 et 1,089 chez les hommes.

**Conclusion:**

Le modèle de l'OMS pour l'Asie orientale a révélé des taux de discrimination modérés en termes de maladies cardiovasculaires au sein de la population chinoise, et ses performances prévisionnelles en la matière sont limitées dans diverses régions du pays. Un recalibrage en fonction des régions a permis d'améliorer considérablement la discrimination et le calibrage pour la population générale.

## Resumen

**Objetivo:**

Validar el modelo de predicción del riesgo de enfermedades cardiovasculares sin pruebas de laboratorio de la Organización Mundial de la Salud (OMS) en regiones de China.

**Métodos:**

Se realizó una validación externa del modelo de la OMS para Asia Oriental a partir del conjunto de datos del China Kadoorie Biobank, un estudio de cohortes en curso con 512 725 participantes seleccionados en 10 regiones de China entre 2004 y 2008. También se volvieron a calcular los parámetros de recalibración para el modelo de la OMS en cada región y se evaluó el rendimiento predictivo del modelo antes y después de la recalibración. Asimismo, se evaluó el rendimiento discriminatorio mediante el índice C de Harrell.

**Resultados:**

Se incluyeron 412 225 participantes de entre 40 y 79 años. Durante una mediana de seguimiento de 11 años, se registraron 58 035 y 41 262 casos nuevos de enfermedades cardiovasculares en mujeres y hombres, respectivamente. El índice C de Harrell del modelo de la OMS fue de 0,682 en mujeres y 0,700 en hombres, pero varió entre regiones. El modelo de la OMS subestimó el riesgo de enfermedad cardiovascular a 10 años en la mayoría de las regiones. Tras la recalibración en cada región, tanto la discriminación como la calibración mejoraron en toda la población. El índice C de Harrell aumentó de 0,674 a 0,749 en las mujeres y de 0,698 a 0,753 en los hombres. Los cocientes entre los casos predichos y los observados antes y después del recalibrado fueron de 0,189 y 1,027 en las mujeres y de 0,543 y 1,089 en los hombres.

**Conclusión:**

El modelo de la OMS para Asia Oriental arrojó una discriminación moderada para las enfermedades cardiovasculares en la población china y tuvo una predicción limitada para el riesgo de enfermedades cardiovasculares en diferentes regiones de China. La recalibración para diversas regiones mejoró considerablemente la discriminación y la calibración en la población general.

## ملخص

**الغرض:**

التحقق من نموذج منظمة الصحة العالمية، غير المعتمد على المختبر، للتنبؤ بمخاطر أمراض القلب والأوعية الدموية في مناطق الصين.

**الطريقة:**

قمنا بإجراء تحقق خارجي من نموذج منظمة الصحة العالمية لشرق آسيا باستخدام مجموعة بيانات بنك كادوري بيوبانك الصيني، وهي دراسة إترابية جارية بمشاركة 512725 مشاركًا تم الاستعانة بهم من 10 مناطق في الصين في الفترة من 2004 إلى 2008. قمنا أيضًا بإعادة حساب معلمات إعادة معايرة نموذج منظمة الصحة العالمية في كل منطقة، وقمنا بتقييم الأداء التنبؤي للنموذج قبل إعادة المعايرة وبعدها. قمنا بتقييم أداء التمييز من خلال مؤشر Harrell's C.

**النتائج:**

قمنا بتضمين 412225 مشاركًا تتراوح أعمارهم بين 40 و79 عامًا. أثناء متوسط متابعة خلال 11 عامًا، تم تسجيل 58035 و41262 حالة إصابة بأمراض القلب والأوعية الدموية في النساء والرجال، على الترتيب. كان مؤشر Harrell's C لنموذج منظمة الصحة العالمية 0.682 في النساء، و 0.700 عند الرجال، ولكنه اختلف بين المناطق. قلل نموذج منظمة الصحة العالمية من تقدير مخاطر الإصابة بأمراض القلب والأوعية الدموية لمدة 10 سنوات في معظم المناطق. بعد إعادة المعايرة في كل منطقة، تحسّن كل من التمييز والمعايرة في إجمالي السكان. زاد مؤشر Harrell’s C من 0.674 إلى 0.749 في النساء، ومن 0.698 إلى 0.753 في الرجال. كانت النسب بين الحالات المتنبأ بها، والحالات التي تمت ملاحظتها قبل إعادة المعايرة وبعدها، 0.189 و1.027 في النساء، و0.543 و1.089 في الرجال.

**الاستنتاج:**

أسفر نموذج منظمة الصحة العالمية لشرق آسيا عن تمييز معتدل لأمراض القلب والأوعية الدموية في السكان الصينيين، وكان له تنبؤ محدود بخطر الإصابة بأمراض القلب والأوعية الدموية في مناطق مختلفة في الصين. أدت إعادة المعايرة للمناطق المختلفة إلى تحسين التمييز والمعايرة بشكل كبير في إجمالي السكان.

## 摘要

**目的:**

验证世界卫生组织 (WHO) 非实验室心血管疾病风险预测模型在中国各地区的性能。

**方法:**

我们利用中国慢性病前瞻性研究 (China Kadoorie Biobank,CBK) 的数据集对东亚的 WHO 模型进行了外部验证，CBK 是一项正在进行的队列研究，于 2004 年至 2008 年从中国 10 个地区招募了 512,725 名参与者。我们还重新计算了 WHO 模型在每个地区的重新校准参数，并评估了模型在重新校准前后的预测性能。我们采用 Harrell 的 C 指数评估了其区分性能。

**结果:**

我们纳入了 412,225 名年龄在 40-79 岁之间的参与者。在中位随访的 11 年期间，分别记录了 58,035 例女性和 41,262 例男性心血管疾病事件。WHO 模型的 Harrell 的 C 指数在女性中为 0.682，在男性中为 0.700，但不同地区有所差异。WHO 模型低估了大多数地区的 10 年心血管疾病风险。在每个地区重新校准模型后，对整个人群的区分度和校准度均有所提高。女性的 Harrell 的 C 指数从 0.674 增加到 0.749，男性则从 0.698 增加到 0.753。重新校准前后，女性预测病例与观察病例的比率分别为 0.189 和 1.027，男性分别为 0.543 和 1.089。

**结论:**

世界卫生组织 (WHO) 东亚模型对中国人群心血管疾病的区分度中等，对中国不同地区心血管疾病风险的预测能力有限。针对不同地区的模型重新校准极大地提高了对整个人群的区分度和校准度。

## Резюме

**Цель:**

Валидировать модель прогнозирования риска развития сердечно-сосудистых заболеваний, разработанную Всемирной организацией здравоохранения (ВОЗ) на основе результатов внелабораторных исследований, в регионах Китая.

**Методы:**

Проведена внешняя валидация модели ВОЗ для стран Восточной Азии с использованием набора данных China Kadoorie Biobank, продолжающегося когортного исследования с 512 725 участниками, набранными из 10 регионов Китая в 2004–2008 гг. Также в каждом регионе был выполнен повторный расчет параметров рекалибровки для модели ВОЗ и оценена прогностическая эффективность модели до и после рекалибровки. Эффективность дискриминации оценивалась с помощью индекса конкордантности Харрелла.

**Результаты:**

В исследование было включено 412 225 участников в возрасте 40–79 лет. В течение медианного периода последующего наблюдения продолжительностью 11 лет было зарегистрировано 58 035 и 41 262 случая развития сердечно-сосудистых заболеваний у женщин и мужчин соответственно. Индекс конкордантности Харрелла для модели ВОЗ составил 0,682 у женщин и 0,700 у мужчин, но различался в разных регионах. В результате применения модели ВОЗ был недооценен 10-летний риск развития сердечно-сосудистых заболеваний в большинстве регионов. После повторной калибровки в каждом регионе были улучшены показатели дискриминации и калибровки в общей популяции. Индекс конкордантности Харрелла увеличился с 0,674 до 0,749 у женщин и с 0,698 до 0,753 у мужчин. Отношение прогнозируемых и наблюдаемых случаев до и после повторной калибровки составило 0,189 и 1,027 у женщин и 0,543 и 1,089 у мужчин.

**Вывод:**

Модель ВОЗ для стран Восточной Азии выявила умеренную дискриминацию в отношении сердечно-сосудистых заболеваний среди населения Китая и сопровождалась ограниченным прогнозированием риска развития сердечно-сосудистых заболеваний в различных регионах Китая. Повторная калибровка по различным регионам значительно улучшила показатели дискриминации и калибровки в общей популяции.

## Introduction

Cardiovascular diseases, including coronary artery disease and stroke, are the leading causes of death and disability worldwide.^1^ Risk prediction models are important tools for identifying high-risk individuals who can benefit from early primary prevention of cardiovascular disease.^2^^–^^6^ Some terminology related to risk prediction models is explained in Box 1.^7^^,^^11^

Box 1 **Terminology for the study of the cardiovascular risk prediction model**

*External validation:* Before a model is widely used, the predictive performance of the model usually needs to be estimated in a population other than the one from which the model was developed; a process called external validation.^7^

*Predictive performance:* The accuracy of the predictions made by a model are expressed in terms of discrimination or calibration.^7^

*Discrimination:* Discrimination performance indicates the ability of the model to distinguish between people who did and did not develop the disease of interest, which is usually assessed by Harrell’s C index.^7^

*Harrell’s C index:* This index estimates the probability of the model correctly predicting who will have a cardiovascular event first in a randomly selected pair of participants.^8^ The value of this index is between 0.5 and 1.0. Generally, a C index above 0.7 indicates a good prediction model.

*Calibration:* Calibration performance indicates the agreement between observed risks and risks predicted by the model, which is usually assessed by the calibration plot.^7^

*Calibration plot:* The mean predicted risks at 10 years with the observed risks across deciles of predicted risks were plotted as a scatter plot. If the observed risks and mean predicted risks agree over the whole range of probabilities, the plot shows a 45-degree line (that is, the slope is 1.0), which indicates ideal calibration performance.

*Ratios of predicted to observed cases:* A ratio of 1.0 indicates perfect calibration; ratios greater than 1.0 indicate overestimation, and those less than 1.0 indicate underestimation.^9^

*Nam-D'Agostino test:* This is a statistical test for quantitative measurement of calibration performance, whereby a smaller *χ^2^* value represents a better calibration performance.^10^

*Recalibration:* When the calibration performance is not good, the model usually needs to be adjusted to the target population (recalibration) to improve its usefulness.^11^

The World Health Organization (WHO) has developed new models to estimate cardiovascular disease risk for people aged 40–80 years in 21 Global Burden of Disease regions, including laboratory-based and non-laboratory-based models.^12^ The non-laboratory-based risk model (hereafter called the WHO model) is more applicable in lower-resource regions where blood-based biomarkers, such as lipid levels, are not widely available for all individuals. The WHO model for East Asia was recommended for predicting individuals’ cardiovascular disease risk in China. However, the model does not take into account important differences in the geographical patterns of incidence, prevalence and mortality of cardiovascular disease (overall and the main subtypes) or the prevalence of the major contributing risk factors for the disease in China.^13^^–^^15^ A previous study conducted an external validation of the WHO model for East Asia among 29 337 participants from 16 provinces of China. The researchers found that the model overestimated the observed cardiovascular disease risk in China; however, the predictive performance of the model in different regions of China was not evaluated.^16^

In the current study we aimed to validate and recalibrate the WHO model for East Asia in a different population of China, using the data set from the China Kadoorie Biobank study which covers 10 diverse regions. We examined regional differences in the incidence of cardiovascular disease in China by comparing the performance of the WHO model in predicting coronary artery disease and stroke in the study population, before and after separate recalibration in the 10 regions.

## Methods

### Study population

China Kadoorie Biobank is an ongoing prospective study with 512 725 participants aged 30–79 years, enrolled from 10 diverse regions of China (five urban, five rural) starting in 2004–2008. Details of the study have been described elsewhere^17^^,^^18^ and in the authors’ online repository.^19^ Briefly, the baseline questionnaire collected information on participants’ sociodemographic characteristics, lifestyle behaviours, dietary habits, personal health (including self-reported histories of coronary artery disease, stroke and transient ischaemic attacks) and family medical history. A 10 mL random blood sample was collected for each participant, with the time of the last meal recorded.

**Fig. 1:**
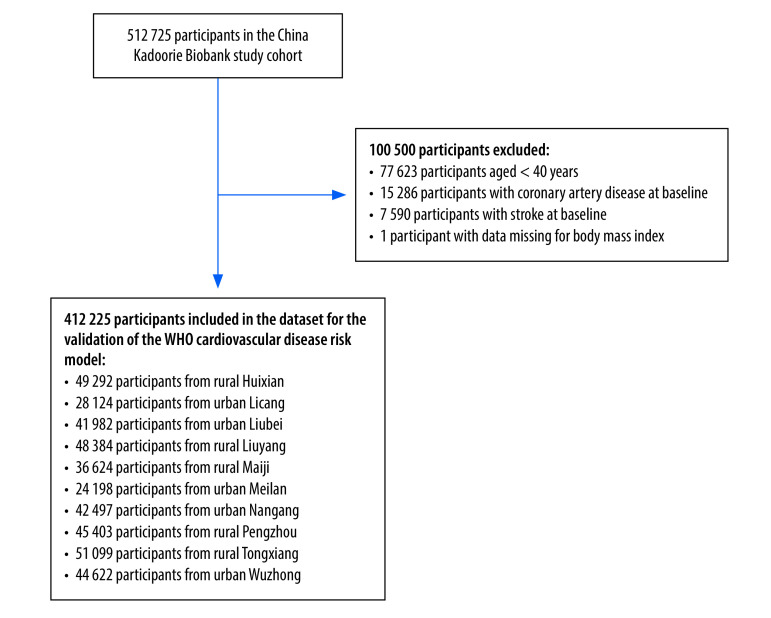
Flowchart in the validation of the WHO non-laboratory-based cardiovascular disease risk prediction model in 10 regions of China

For the present analysis, conducted in May 2022, we excluded participants who were younger than 40 years old (77 623 people), those with a history of coronary artery disease (15 286 people) or stroke or transient ischaemic attack (7590 people), and those who had missing data on body mass index (one person) at the baseline survey. We therefore included a total of 412 225 participants (Fig. 1).

WHO: World Health Organization.

Note: Detailed geographical information about each study region are in the authors’ online repository.^19^

We obtained ethical approval from the ethical review committee of the Chinese Center for Disease Control and Prevention in Beijing, China, and the Oxford tropical research ethics committee, University of Oxford, United Kingdom of Great Britain and Northern Ireland. All participants provided a written informed consent form.

### Data collection 

The variables we used included sex, age, smoking status, systolic blood pressure and body mass index, all of which are risk predictors in the WHO non-laboratory-based cardiovascular disease model.^12^ Details on the collection and definition of each predictor have been described in our previous study.^20^

We followed up all participants to determine any cardiovascular disease events experienced since their baseline enrolment. These incident events were identified from local disease and death registries and the national health insurance system, or by directly contacting the participants.^17^ A total of 500 029 (97.5%) participants were linked to the Chinese health insurance system. Only 4009 participants (1.0%) were lost to follow-up before the date of the end of follow-up (31 December 2017). Since the period from the baseline survey to the date of loss to follow-up could still be included in analyses, we did not exclude these participants. We used information from records of underlying and multiple causes of death and from primary and secondary diagnoses at discharge from hospital. Trained staff of the China Kadoorie Biobank research team (who were blinded to the baseline information) coded all cardiovascular events using the International Statistical Classification of Diseases and Related Health Problems, 10th revision (ICD-10). The medical records of the first event were retrieved and reviewed by specialist physician adjudicators.^21^ By October 2018, of the retrieved medical records of 33 515 coronary artery disease cases (ICD-10 codes: I20–I25), 34 758 ischaemic stroke cases (code: I63), and 5023 haemorrhagic stroke cases (codes: I60–I61), the number of confirmed cases was 29 448 (87.9%), 31 806 (91.5%), and 4041 (80.4%), respectively.

We included only the first cardiovascular disease event during follow-up, unless otherwise specified. For example, if a participant was recorded with both coronary artery disease and stroke (simultaneously or sequentially), we used the date of the first of these two events in the analysis of all types of cardiovascular disease. When coronary artery disease or stroke was analysed as different outcomes, we considered the dates of the first coronary artery disease event and first stroke event separately.

### Outcome definitions

**Fig. 2:**
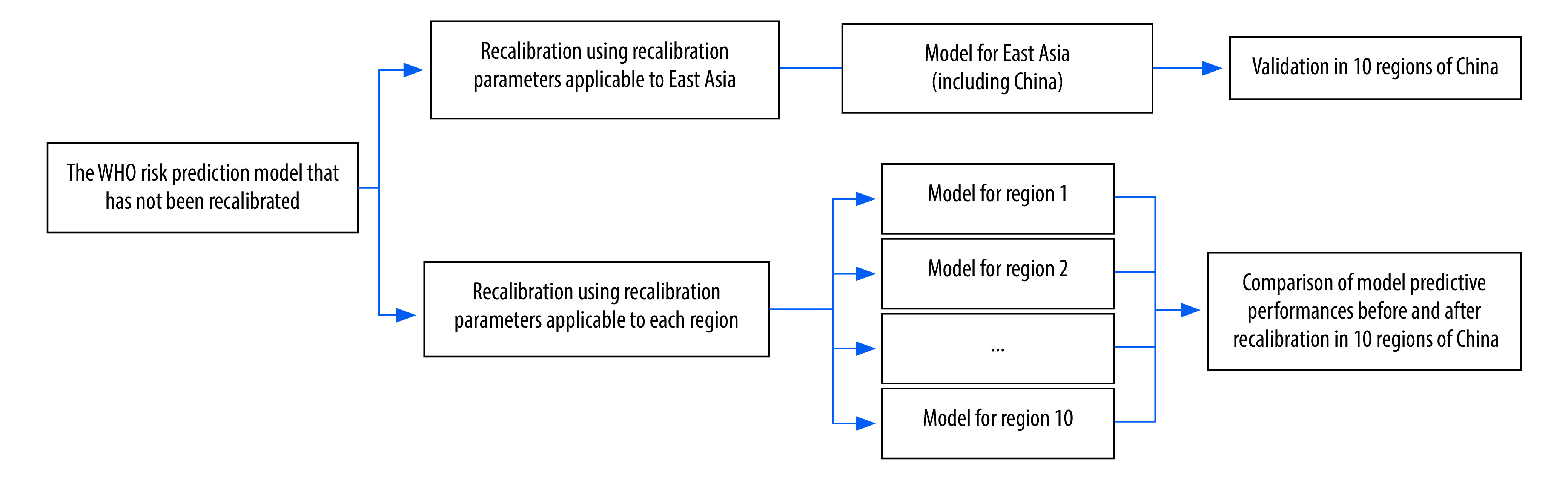
Study design in the validation of the WHO non-laboratory-based cardiovascular disease risk prediction model in 10 regions of China

The developers of the WHO model recalibrated the original model in 21 Global Burden of Disease regions to adapt the model to the circumstances of different regions. Different data sets were used when developing and recalibrating the model. The definitions of cardiovascular disease outcomes (defined using ICD-10 codes) in the process of deriving the model were narrower than those in the process of recalibrating it.^1^^,^^12^ We used both definitions in the present study.^19^ Briefly, in the first component of the study (Fig. 2), the definition was the same as the Global Burden of Disease study in 2017, one of the data sources to calculate the recalibration parameters for the WHO model.^1^ This makes the model we validated consistent with the model recommended by WHO.^2^ In the second component of the study, the definition was the same as the definition used to derive the WHO model, so that the outcome definitions of model recalibration and model derivation were consistent.^12^

WHO: World Health Organization.

Note: More details of the study design are in the authors’ online repository.^19^

### Statistical analysis

We conducted all analyses separately for women and men. Briefly, we first calculated the 10-year risk of cardiovascular disease for each participant according to the WHO uncalibrated model.^12^ Subsequently, in the first component of the study, we recalibrated the calculated risks according to the latest recalibration parameters in 2017 applicable to East Asia (WHO model for East Asia).^12^ In the second component of the study, we recalibrated the calculated risks in the 10 study regions of China (WHO model for each region). More details are in the online repository.^19^^,^^22^

We evaluated the discrimination and calibration performance of the WHO model before and after recalibration in each study region and across the overall study population. We assessed discrimination performance by Harrell’s C index.^8^ We assessed calibration performance graphically by comparing the mean predicted risks at 10 years with the observed risks across deciles of predicted risks in the calibration plot. The observed 10-year risks were estimated using the Kaplan–Meier method.^23^ We calculated the ratios of predicted to observed cases.^9^ We used the Nam-D’Agostino test to quantify the agreement or fit (Box 1).^10^

We conducted the following sensitivity analyses. First, because the China Kadoorie Biobank cohort was started in 2004–2008, we used recalibration parameters in 2005, 2010, and 2015 that were derived by the developers of the WHO model to recalibrate the WHO model.^19^ Second, due to the higher incidence of stroke in China compared with high-income countries, applying the outcome definition used in the derivation process of the WHO model could lead to an artificially low proportion of coronary artery disease in total cardiovascular disease. Therefore, in the second component of this study, we instead adopted the outcome definition of the China Kadoorie Biobank model, which is a previously developed non-laboratory-based risk prediction model and has a broader definition of coronary artery disease and a narrower definition of stroke than the WHO model.^20^ More details are in the online repository.^19^

The study adhered to the TRIPOD (Transparent Reporting of a multivariable prediction model for Individual Prognosis Or Diagnosis) statement for reporting.^19^^,^^24^ We conducted analyses using Stata, version 17.0 (Stata Corp., College Station, United States of America). The figures were produced using R, version 3.6.0 (R Foundation for Statistical Computing, Vienna, Austria).

## Results

### Study population

A total of 412 225 participants from 10 different regions of China were included in the current study: 241 556 (58.6%) were female and 230 802 (56.0%) were rural residents. The mean age was 54.3 years (standard deviation: 9.2; Table 1). There were substantial differences in the levels of cardiovascular disease risk factors among the 10 study regions. For example, the age-adjusted proportion of daily smokers in Meilan was 0.2% among women and 39.2% among men, while the corresponding proportions in Pengzhou were 10.2% and 64.7%, respectively (full data are in the online repository).^19^

**Table 1:** Selected characteristics of the China Kadoorie Biobank study participants, by sex

Variable	Women	Men
**Baseline characteristics**		
Total no. of participants	241 556	170 669
No. (%) of participants living in rural areas	133 155 (55.1)	97 647 (57.2)
Age years, median (25–75th percentile)	52.8 (46.2–59.9)	54.0 (47.4–62.0)
No. (%) of participants with primary school education or below	145 314 (60.2)	78 040 (45.7)
No. (%) of participants with annual household income < 10 000 Renminbi	71 431 (29.6)	45 286 (26.5)
No. (%) of participants that were current daily smokers	5 602 (2.3)	97 132 (56.9)
No. (%) of participants using blood pressure-lowering treatment	33 332 (13.8)	19 800 (11.6)
No. (%) of participants with diabetes, self-reported	8 128 (3.4)	4 876 (2.9)
No. (%) of participants with diabetes, all^a^	15 700 (6.5)	9 732 (5.7)
Systolic blood pressure, median (25–75th percentile) mmHg	128.5 (116.0–144.0)	130.5 (119.5–144.5)
Diastolic blood pressure, median (25–75th percentile) mmHg	76.5 (70.0–84.0)	79.0 (71.5–86.5)
Body mass index, median (25–75th percentile) kg/m^2^	23.7 (21.5–26.1)	23.1 (21.0–25.5)
Waist circumference, median (25–75th percentile) cm	79.0 (72.8–85.6)	81.3 (74.3–88.5)
**Follow-up period** ^b^		
Total person-years observed	2 642 595	1 805 894
Follow-up time, median (25–75th percentile) years	11.1 (10.2–12.1)	11.0 (10.0–12.0)
No. (%) of participants with 10 or more years of follow-up	198 767 (82.3)	130 806 (76.6)
**Cardiovascular disease outcomes at follow-up, no. of incident cases^c^**		
Outcome definition in the WHO model recalibration process^1^^,d^		
Coronary artery disease	24 403	16 696
Stroke	43 351	30 778
Total	58 035	41 262
Outcome definition in the WHO model derivation process^12^		
Coronary artery disease	3 835	4 689
Stroke	40 171	29 241
Total	42 919	32 658
Outcome definition in the China Kadoorie Biobank model derivation process^20^^,e^		
Coronary artery disease	24 403	16 696
Stroke	27 222	22 818
Total	45 200	34 798

WHO: World Health Organization.

^a^ We defined all diabetes as self-reported diabetes or screening-detected diabetes, defined as (i) random blood glucose ≥ 7.0 mmol/L and fasting time ≥ 8 hours, (ii) random blood glucose ≥ 11.1 mmol/L and fasting time < 8 hours or (iii) fasting blood glucose ≥ 7.0 mmol/L on subsequent testing.

^b^ We calculated person-year as the time from the baseline date to the date of death, loss to follow-up, or end of follow-up (31 December 2017), whichever came first.

^c^ We applied different definitions of cardiovascular disease to different components of the study. We included only the first cardiovascular disease event. If a participant was recorded with a coronary artery disease event and a stroke event successively or simultaneously the person was counted as having one cardiovascular disease event, one coronary artery disease event, and one stroke event. Detailed definitions of each outcome are in the online repository.^19^

^d^ Outcome definition consistent with the Global Burden of Disease Study 2017.^1^

^e^ Outcome definition used in the sensitivity analysis.^20^

Note: WHO model refers to the World Health Organization non-laboratory-based cardiovascular disease risk prediction model.^12^

### Cardiovascular cases

During a median follow-up of 11 years, we recorded 58 035 and 41 262 new cardiovascular disease cases (defined according to WHO recalibration criteria) among women and men, respectively. The number of coronary artery disease cases according to the definition in the WHO model derivation process (non-fatal, ICD-10 codes: I21–I23 and fatal, codes: I21–I25) was far fewer than that according to the definition in the China Kadoorie Biobank model derivation process (any code: I20–I25; Table 1). 

There were large variations in the 10-year risk of cardiovascular disease overall, and its subtypes, among the 10 study regions. For example, the 10-year risk of stroke according to the definition in the WHO model derivation process (any ICD-10 code: I60–I69) was over 30% in both sexes in Nangang, but lower than 10% in Wuzhong.^19^

### Validation of model

**Fig. 3:**
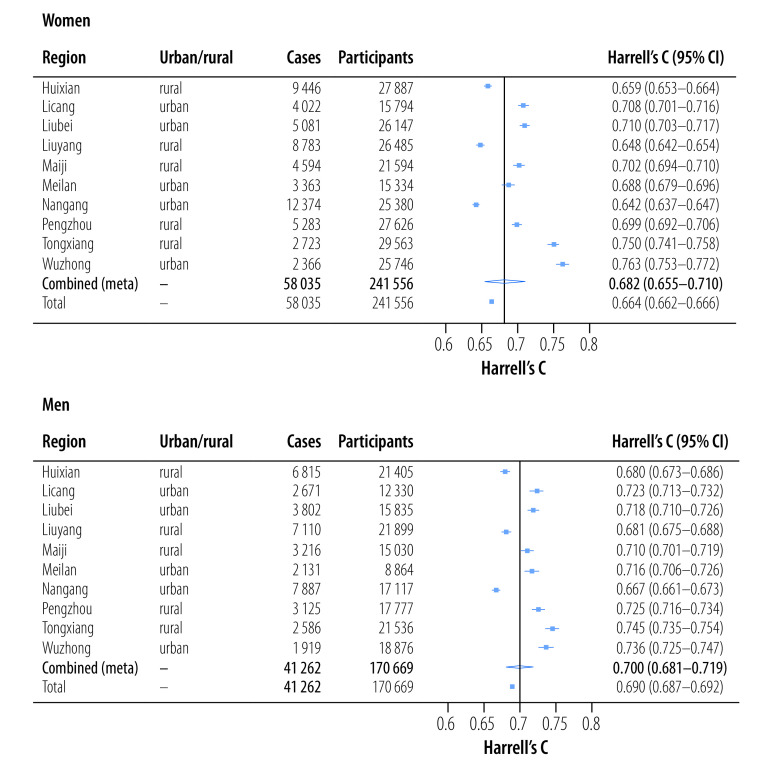
Discrimination performance of the WHO non-laboratory cardiovascular disease risk model for East Asia in 10 regions of China

CI: confidence interval; NA: not applicable; WHO: World Health Organization.

Notes: The column Cases represents the number of incident cardiovascular diseases in each study region. We defined cardiovascular disease according to the definition used by the recalibration process of the WHO non-laboratory cardiovascular disease risk model. The recalibration parameters applied to China in 2017 were used for the recalibration of the WHO model. Harrell’s C index estimates the probability of the model correctly predicting who will have a cardiovascular disease event first in a randomly selected pair of participants. The value of this index is between 0.5 and 1.0. Generally, a C index above 0.7 indicates a good prediction model. We calculated the C index of the total row based on the whole sample, ignoring the region. The vertical line represents the combined C index, which is the sum of the C index of each study region weighted by inverse variance (meta-analysis). We calculated the 95% CI of the combined C index by using the *t* distribution with nine degrees of freedom. More details of the methods are in the authors’ online repository.^19^

In the external validation of the WHO model for East Asia, Harrell’s C index was 0.682 (95% confidence interval, CI: 0.655–0.710) among women, with substantial variation among regions, indicating moderate discrimination performance (Fig. 3). The C index was lowest in Nangang (0.642; 95% CI: 0.637–0.647) and highest in Wuzhong (0.763; 95% CI: 0.753–0.772). C indices among men were similar to those among women. As for the calibration performance, the WHO model for East Asia underestimated the 10-year risk of cardiovascular disease for the overall population and all study regions except Wuzhong and Tongxiang.^19^ After recalibrating the WHO model with recalibration parameters derived in different years, the discrimination performance barely changed,^19^ and the underestimation of the 10-year risk of cardiovascular disease persisted.^19^

### Recalibration of model

After recalibrating the WHO model in each study region, we found that the discrimination performance of the WHO model was improved in the overall study population. Among women, Harrell’s C index increased by 0.075 from 0.674 (95% CI: 0.672–0.677) to 0.749 (95% CI: 0.746–0.751). Among men, the C index increased from 0.698 (95% CI: 0.695–0.701) to 0.753 (95% CI: 0.750–0.755), an increment of 0.055. However, recalibration had little effect on the discrimination performance in each study region. For example, among men in Wuzhong, where the change in C index after recalibration was the largest, the C index increased from 0.740 (95% CI: 0.728–0.752) to 0.747 (95% CI: 0.735–0.759), an increment of 0.007 (Table 2).

**Table 2:** Discrimination performance of the WHO non-laboratory cardiovascular disease risk model, before and after recalibration, in 10 regions of China

Region	Area	Women				Men		
		No. of incident cases of cardiovascular disease	Harrell’s C (95% CI)			No. of incident cases of cardiovascular disease	Harrell’s C (95% CI)	
			Before recalibration^a^	After recalibration			Before recalibration^a^	After recalibration
Huixian	Rural	7 222	0.667 (0.661–0.673)	0.668 (0.661–0.674)		5 594	0.683 (0.676–0.690)	0.689 (0.682–0.696)
Licang	Urban	1 439	0.768 (0.756–0.779)	0.767 (0.755–0.779)		1 457	0.747 (0.734–0.759)	0.752 (0.740–0.764)
Liubei	Urban	3 518	0.725 (0.717–0.734)	0.725 (0.716–0.733)		2 867	0.729 (0.720–0.738)	0.734 (0.725–0.742)
Liuyang	Rural	7 263	0.643 (0.636–0.649)	0.643 (0.636–0.649)		5 870	0.678 (0.671–0.685)	0.684 (0.677–0.691)
Maiji	Rural	3 779	0.719 (0.711–0.727)	0.720 (0.712–0.728)		2 724	0.728 (0.718–0.738)	0.732 (0.723–0.742)
Meilan	Urban	2 913	0.692 (0.683–0.701)	0.691 (0.682–0.700)		1 850	0.718 (0.707–0.729)	0.722 (0.711–0.733)
Nangang	Urban	9 290	0.654 (0.648–0.659)	0.654 (0.648–0.659)		6 352	0.680 (0.673–0.687)	0.682 (0.676–0.689)
Pengzhou	Rural	3 620	0.699 (0.691–0.708)	0.700 (0.692–0.709)		2 373	0.730 (0.720–0.740)	0.736 (0.726–0.746)
Tongxiang	Rural	2 035	0.756 (0.746–0.767)	0.756 (0.745–0.766)		2 024	0.747 (0.736–0.757)	0.752 (0.741–0.762)
Wuzhong	Urban	1 840	0.762 (0.751–0.773)	0.761 (0.750–0.771)		1 547	0.740 (0.728–0.752)	0.747 (0.735–0.759)
Combined (meta)^b^	NA	42 919	0.690 (0.660–0.721)	0.690 (0.660–0.720)		32 658	0.706 (0.686–0.727)	0.711 (0.691–0.732)

CI: confidence interval; NA: not applicable; WHO: World Health Organization.

^a^ Since the outcome definition here is different from that in Fig. 3, there are some differences between the C indices before recalibration and those in Fig. 3.

^b^ The combined C index was the sum of the C index of each study region weighted by inverse variance (meta-analysis). We calculated the 95% CI of the combined C index by using the *t* distribution with nine degrees of freedom.

Notes: We defined cardiovascular disease according to the definition used by the derivation process of the WHO non-laboratory cardiovascular disease risk model. We recalibrated the WHO model in each study region. More details of the methods are in the authors’ online repository.^19^ Harrell’s C index estimates the probability of the model correctly predicting who will have a cardiovascular disease event first in a randomly selected pair of participants. The value of this index is between 0.5 and 1.0. Generally, a C index above 0.7 indicates a good prediction model.

**Fig. 4:**
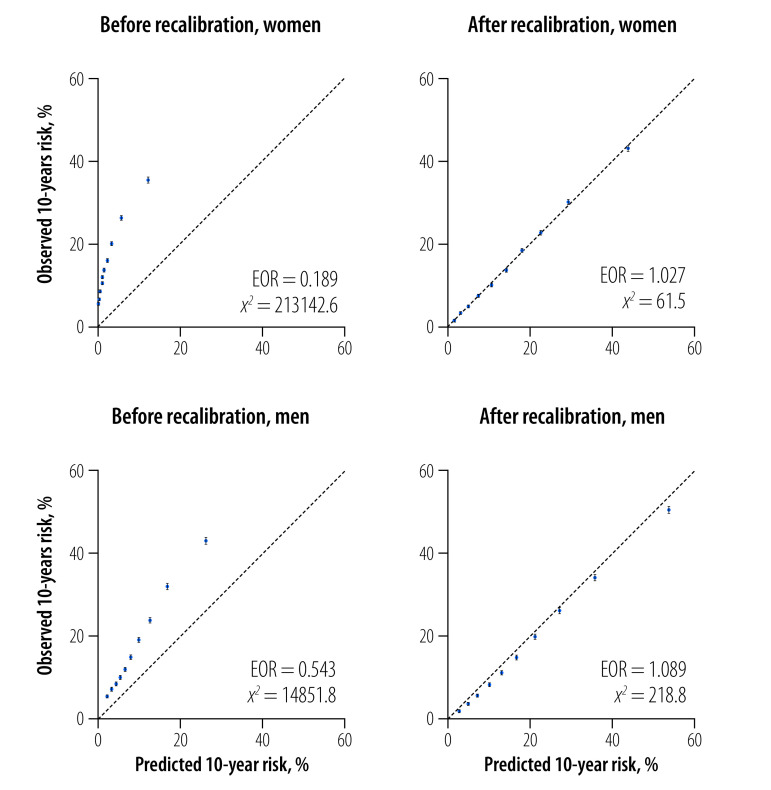
Predictive performance of the WHO cardiovascular disease risk model before and after recalibration, in 10 regions of China

EOR: ratios of predicted to observed events; WHO: World Health Organization.

Notes: The closer the points are to the diagonal dotted line (the 45 degree line)  the better the calibration performance is. When the point is above the dotted line  it indicates that the model underestimates the actual risk; conversely, it indicates that the model overestimates the actual risk. A ratio of 1.0 indicates perfect calibration; ratios greater than 1.0 indicate overestimation  and those less than 1.0 indicate underestimation. The *χ^2^*-values represent the Nam-D’Agostino test with nine degrees of freedom. A smaller *χ^2^*-value represents a better calibration performance. We defined cardiovascular disease events according to the definition used by the derivation process of the WHO cardiovascular disease risk model. We recalibrated the WHO model in each study region. More details of the methods are in the authors’ online repository.^19^

The calibration performance of the recalibrated WHO model was close to 1.0, the ideal level, in the overall study population (Fig. 4) and in the 10 study regions.^19^ After recalibration, the discrimination performance of the WHO model was improved in older people (≥ 65 years) and individuals with hypertension, diabetes, low education level and low household income.^19^ The recalibrated WHO model was well calibrated in older people and those with hypertension, but slightly underestimated the risk of cardiovascular disease in people with diabetes.^19^

When we instead adopted the disease outcome definition of the China Kadoorie Biobank model, the discrimination and calibration performance was improved in the overall study population.^19^ However, the recalibrated model still slightly underestimated the 10-year risk of cardiovascular disease in participants with diabetes.^19^

## Discussion

We found that the overall discrimination of the WHO model was moderate, and the 10-year cardiovascular disease risk of the China Kadoorie Biobank study participants was underestimated in most regions. After recalibration of the WHO model in each study region, the discrimination and calibration performances of the model were greatly improved in the overall study population.

The pooled Harrell’s C index of the WHO model for East Asia was only about 0.7 in both sexes, which is lower than in previous studies conducted in Chinese populations. In an external validation study based on the Asia Pacific Cohorts Studies Collaboration and the China Multi-Provincial Cohort Study, the pooled C index of the non-laboratory-based WHO model for East Asia was 0.741 (95% CI: 0.725–0.757).^12^ When applying the WHO model in the Prediction for Atherosclerotic Cardiovascular Disease Risk in China cohort, the C index was 0.754 (95% CI: 0.731–0.777) in women and 0.762 (95% CI: 0.744–0.781) in men.^16^ Differences in the definition of outcomes and in the study population could have influenced the discrimination performance. In the present study, we adopted the same definition of disease outcomes that was used in the recalibration process of the WHO model. This definition includes non-fatal angina (ICD-10 code: I20) for classifying coronary artery disease, and other cerebrovascular diseases (code: I65–I69) for classifying stroke.^1^ Our definition is broader than that adopted by the previous studies in China.^12^^,^^16^ These differences could partly explain the overestimation of cardiovascular disease risk in the external validation of the Prediction for Atherosclerotic Cardiovascular Disease Risk in China project.^16^

The WHO model for East Asia underestimated the cardiovascular disease risk to a variable extent in most study regions. Separate recalibration of the WHO model in each region achieved almost ideal calibration performance. In other words, the observed risks and risks predicted by the model were similar. The findings suggest a universal model is unsuitable for direct application to different regions in China due to large regional differences in the incidence of cardiovascular disease subtypes. Models need to be recalibrated according to the local prevalence of cardiovascular disease risk factors and disease incidence rates before being applied to a specific region. The Prediction for Atherosclerotic Cardiovascular Disease Risk in China study did not evaluate the calibration performance of the WHO model by region, thus making the conclusions less reliable than in the current study.^16^ However, in our study, participants of each study region came from a relatively small geographical area in China. It is not feasible to update the model across the whole country according to the current regional size. A possible approach is first to update the model in a larger area, such as a province, and then to update the model in smaller geographical areas. External validation studies conducted in other regions of China are needed to examine our findings.

Recalibration significantly improved the discrimination of the WHO model in the overall population, highlighting the importance of recalibration in different regions of China. Recalibration by region is equivalent to adding the region as a predictor. Due to the significant differences in the spatial patterns of incidence of cardiovascular disease and the prevalence of major cardiovascular disease risk factors in China,^13^^–^^15^ we observed a significant improvement in discrimination in the overall population. However, recalibration had little impact on discrimination in each study region. Since recalibration changed the predicted risk but not the order of predicted risk for each participant,^25^ both the discrimination of the coronary artery disease and the stroke submodels remained unchanged in each study region. Therefore, the discrimination performance of the total cardiovascular disease model was not greatly affected.

The differences in discrimination performance of the WHO model among the 10 study regions persisted after recalibration and could not be explained by the spatial patterns mentioned above. China is a large, rapidly developing upper-middle-income country, where cardiovascular disease risk factors might differ from the well-established risk factors in high-income countries. For example, risk factors such as environmental hazards in the home, work and broader outdoor environment might also influence the incidence of cardiovascular disease in China. These risk factors were not included in the current model and might affect the discrimination of the model to varying degrees in different regions. The discrimination of the WHO model was not good (C index < 0.7) in some study regions, such as Huixian, Liuyang and Nangang. There may be specific risk factors in these regions that need to be determined. However, other known cardiovascular disease risk factors might help improve risk prediction. Specifically, the current model could be used to screen a subgroup of people with a relatively high risk of cardiovascular disease; subsequently, other cardiovascular disease risk factors could be evaluated in selected populations. Among non-laboratory-based indicators, previous studies have found that waist circumference was a better predictor of cardiovascular disease than body mass index, and that including antihypertensive treatment in the model improves risk prediction.^20^^,^^26^ Measuring diastolic blood pressure, level of education, waist-to-hip ratio in men and physical activity level in women could improve the risk prediction of haemorrhagic stroke in the China Kadoorie Biobank study.^20^ Other physical examination indicators such as ankle–brachial index and arterial stiffness, psychosocial and work stress, and environmental exposure would also be expected to improve risk prediction.^3^^,^^4^ However, these indicators are not easily available and measurable, limiting their possible application.

We adopted different definitions of cardiovascular disease outcomes in the recalibration process of the WHO model, but the calibration performance of the recalibrated WHO model approached the ideal level regardless of the definition used. These findings suggest that outcome definitions adopted by the model in the practical application could have differed from those used in the model derivation process, indicating the flexibility of the recalibration method proposed by developers of the WHO model.^12^ The main factor affecting the calibration performance of the recalibrated model is more likely to be the reliability of the data source used to generate the recalibration parameters. However, different outcome definitions affect the interpretation of the model. The ratio of the incidence of stroke to incidence of coronary artery disease is higher in China than in high-income countries.^12^ The WHO model adopted a narrower definition of coronary artery disease and a broader definition of stroke than the China Kadoorie Biobank definition. When adopting the outcome definition in the derivation data set of the WHO model, the model mainly predicts the risk of stroke in the present population. In addition, major coronary events – the definition used in the WHO model derivation process – are well defined and measured consistently across studies. However, this narrower definition might underestimate the overall coronary artery disease burden. Currently, there is no consensus on the definition of the disease outcomes for use in cardiovascular disease risk prediction models. Different definitions of outcome have different implications in different contexts: public health, health economics or society in general. Future studies need to determine the most appropriate outcome definition for the context.

The advantages of the present study are that it provided a large external validation study of the WHO model in the Chinese population, with good coverage of regions with different burdens of cardiovascular disease subtypes. All 10 study regions adopted identical procedures and standardized protocols, allowing comparison and pooling of results from the different regions. Less than 1% of China Kadoorie Biobank participants were lost after an average of 11 years of follow-up. There were some limitations to the study, however. First, we were unable to validate the laboratory-based WHO model, since information on blood lipid levels was only available for a subset of participants. Previous studies have suggested the laboratory-based and non-laboratory-based WHO models have similar predictive performances.^12^^,^^16^^,^^27^ However, the model which excludes laboratory biomarkers is more likely to be used in lower-resource regions. Second, the recalibrated WHO model for each study region should be considered a new model. External validation studies are warranted before the model is applied. Third, like most large-scale cohorts, the participants recruited at baseline were volunteers willing to participate in the study. However, the selection bias caused by the loss of follow-up is very small in the China Kadoorie Biobank study. Fourth, the current analyses included only inpatient events, which mainly correspond to more severe conditions. Participants with low socioeconomic status might have delayed hospital visits, which could narrow the difference in hospital visits among groups with different socioeconomic statuses. However, our recalibration significantly improved the discrimination among participants with low socioeconomic status.^19^

Based on a large population-based cohort of Chinese adults, we found that the WHO cardiovascular risk prediction model for East Asia, using non-laboratory-based parameters, was not directly applicable to different regions of China. The model needs to be recalibrated before being used in a specific region in China. In future, to generate parameters for model recalibration, surveillance systems for cardiovascular diseases and risk factors need to be established in different regions of China.
